# Irreversible Denaturation of Maltodextrin Glucosidase Studied by Differential Scanning Calorimetry, Circular Dichroism, and Turbidity Measurements

**DOI:** 10.1371/journal.pone.0115877

**Published:** 2014-12-30

**Authors:** Megha Goyal, Tapan K. Chaudhuri, Kunihiro Kuwajima

**Affiliations:** 1 Okazaki Institute for Integrative Bioscience and Institute for Molecular Science, National Institutes of Natural Sciences, 5-1 Higashiyama, Myodaiji, Okazaki, Aichi, 444-8787, Japan; 2 School of Biological Sciences, Indian Institute of Technology Delhi, Hauz Khas, New Delhi, 110016, India; 3 Department of Functional Molecular Science, School of Physical Sciences, the Graduate University for Advanced Studies (Sokendai), 5-1 Higashiyama, Myodaiji, Okazaki, Aichi, 444-8787, Japan; 4 The Center for the Promotion of Integrated Sciences (CPIS), the Graduate University for Advance Studies (Sokendai), Shonan Village, Hayama, Kanagawa, 240-0193, Japan; 5 School of Computational Sciences, Korea Institute for Advanced Study (KIAS), Dongdaemun-gu, Seoul, 130-722, Korea; Universidad de Granada, Spain

## Abstract

Thermal denaturation of *Escherichia coli* maltodextrin glucosidase was studied by differential scanning calorimetry, circular dichroism (230 nm), and UV-absorption measurements (340 nm), which were respectively used to monitor heat absorption, conformational unfolding, and the production of solution turbidity. The denaturation was irreversible, and the thermal transition recorded at scan rates of 0.5–1.5 K/min was significantly scan-rate dependent, indicating that the thermal denaturation was kinetically controlled. The absence of a protein-concentration effect on the thermal transition indicated that the denaturation was rate-limited by a mono-molecular process. From the analysis of the calorimetric thermograms, a one-step irreversible model well represented the thermal denaturation of the protein. The calorimetrically observed thermal transitions showed excellent coincidence with the turbidity transitions monitored by UV-absorption as well as with the unfolding transitions monitored by circular dichroism. The thermal denaturation of the protein was thus rate-limited by conformational unfolding, which was followed by a rapid irreversible formation of aggregates that produced the solution turbidity. It is thus important to note that the absence of the protein-concentration effect on the irreversible thermal denaturation does not necessarily means the absence of protein aggregation itself. The turbidity measurements together with differential scanning calorimetry in the irreversible thermal denaturation of the protein provided a very effective approach for understanding the mechanisms of the irreversible denaturation. The Arrhenius-equation parameters obtained from analysis of the thermal denaturation were compared with those of other proteins that have been reported to show the one-step irreversible thermal denaturation. Maltodextrin glucosidase had sufficiently high kinetic stability with a half-life of 68 days at a physiological temperature (37°C).

## Introduction

Differential scanning calorimetry (DSC) is a powerful technique for studying thermal denaturation of globular proteins, and the methods for analysis of reversible thermal denaturation have been well elaborated [1]–[4]. However, there are many proteins that undergo irreversible thermal denaturation, and DSC can also be used effectively for the analysis of the irreversible thermal denaturation, although the analysis method is very different from that of the equilibrium denaturation and based on the kinetics of the Arrhenius equation [5]–[11]. Over the last 25 years, a large number of *in vitro* and irreversible, protein thermal denaturation processes have thus been reported, and for many of these proteins, the irreversible denaturation can be described well using a simple phenomenological one-step denaturation model [5]–[11]:
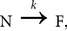
(1)where N and F represent the native and irreversibly denatured state, respectively, and *k* is a temperature-dependent first-order rate constant. The half-life *τ*_1/2_ of the denaturation reaction is given by *τ*_1/2_ = (ln 2)/*k*, and the *τ*_1/2_ value at a physiological temperature (37°C) provides a measure of the kinetic stability of proteins. The kinetic stability of a natural globular protein is often longer than a month or even a year [8], [11], and it is important for understanding protein stability *in vivo* [7], [8], [12], the molecular mechanisms of protein misfolding diseases [9], [11], [13], and natural selection for stability during protein evolution [14]–[16].

The irreversibility of thermal denaturation has often been ascribed to the occurrence of “side” processes such as aggregation [17]–[20], proteolysis [12], [21], or chemical alterations of amino acid residues during the denaturation process [8], [22]–[26], and the heat-induced aggregation has been considered to be a major cause of the irreversibility of thermal denaturation of proteins. However, the molecular details of denaturation and the interrelationship between aggregation and denaturation are not yet well understood. For some proteins, the aggregation takes place concurrently with conformational unfolding in the irreversible thermal denaturation [18]–[20], but for many proteins, the aggregation takes place at a higher temperature after the thermally-induced conformational unfolding, and this aggregation usually accompanies an exothermal effect and sometimes results in formation of precipitates [26]–[31]. The thermal aggregation of globular proteins is usually not represented by a simple single-step process, but it may be assumed that the final aggregated entities are formed via starting assemblies [32]–[37]. The interrelationship between these processes and the irreversible thermal denaturation seems crucial for elucidating the molecular mechanisms of the thermal denaturation of proteins, but this interrelationship has been studied only for a very limited number of proteins [32]–[37]. The interrelationship between aggregation and denaturation may also be important for understanding the mechanisms of human-disease-related amyloidogenesis of proteins, because the amyloidogenesis is brought about by partial denaturation followed by protein aggregation [13], [38].

The present paper deals with the thermal denaturation of maltodextrin glucosidase (MalZ), which is a cytoplasmic enzyme of *Escherichia coli* (*E. coli*) [39], consisting of 604 amino-acid residues with a molecular weight of 69 kDa. MalZ removes a glucose residue from the reducing end of maltodextrin [39], and also exhibits transglycosylation activities [40]. The thermal denaturation of MalZ was studied by DSC, circular dichroism (CD) and UV absorption (turbidity) measurements, which were respectively used to monitor heat absorption, conformational unfolding, and the turbidity production. Although the physical parameters monitored by these measurements were different, the thermal transitions thus obtained were in excellent agreement with each other, and well represented by the one-step irreversible model (Eq. (1)) with a denaturation activation energy of 571 kJ/mol and a *τ*_1/2_ value (37°C) of 68 days. The thermal transitions measured at different protein concentrations by DSC and UV-absorption gave the same Arrhenius-equation parameters, indicating the lack of a protein concentration effect on the thermal denaturation.

From the above results, the thermal denaturation of MalZ was rate-limited by conformational unfolding, which was followed by rapid irreversible formation of aggregates that produced the solution turbidity. This model of the MalZ denaturation is thus entirely consistent with the Lumry-Eyring model of the irreversible thermal denaturation of proteins [41], [42] (see below). The thermal transitions measured by the solution turbidity exhibited typical sigmoidal transition curves, coincident with those measured by DSC and CD, and the turbidity values in a post-transition region were weakly dependent on temperature and independent of the scan rate. It is thus strongly suggested that the irreversibly formed aggregates of MalZ have a definite size and a definite number of protein molecules, which remain preserved during the thermal denaturation. Together, these results show that turbidity measurements together with DSC and CD measurements of the irreversible thermal denaturation of proteins provide a very effective approach for investigating the mechanisms of the irreversible denaturation of proteins. Further studies in this direction may be required to fully elucidate the irreversible thermal denaturation of proteins.

## Materials and Methods

### Materials

The BL21 *E. coli* strain was used for expression of MalZ. The plasmid (pCS19MalZ), which contains the (His)_6_ *malZ* gene, was a generous gift from W. Boos (University of Konstanz, Germany) [43]. All the chemicals used in the study were purchased from Sigma Chemicals Co., and were of the highest purity grade. Double distilled or Milli-Q (Merck Millipore) water was used throughout.

### Protein purification

*E. coli* (BL21(DE3)) cells, harboring pCS19MalZ and overexpressing the protein, were pelleted through centrifugation at 10,000 rpm for 50 min. The supernatant obtained after centrifugation was discarded, and the cell pellet was washed and resuspended in 20 mM sodium phosphate buffer (pH 7.4) containing 500 mM NaCl, a pinch of DNAse I (1 µl/ml of cell lysate), 0.5 mM MgCl_2_ and 1 mM PMSF. The cells were disrupted through sonication, and the supernatant, separated by centrifugation of the lysate, was further purified by Ni^2+^-affinity chromatography. Before loading the supernatant, a column filled with the Ni^2+^-NTA resin was first washed with binding buffer for equilibration. The supernatant was then loaded on the column, and the column was washed with five column volumes of washing buffer. The purified protein was eluted with five column volumes of elution buffer. The buffer compositions used were: (1) 20 mM sodium phosphate, containing 500 mM NaCl, at pH 7.4 for the binding buffer; (2) 20 mM sodium phosphate, containing 500 mM NaCl and 10 mM imidazole, at pH 7.4 for the washing buffer; and (3) 20 mM sodium phosphate, containing 500 mM NaCl and 500 mM imidazole, at pH 7.4 for the elution buffer.

The molar concentrations of purified MalZ used in the following DSC, CD and UV-absorption measurements were determined spectrophotometrically using a molar extinction coefficient of 1.523×10^5^ M^−1 ^cm^−1^, which was estimated from the amino-acid sequence of MalZ [39], [44], [45]; we used the ProtParam tool of ExPAsy (http://web.expasy.org/protparam/).

### DSC measurements

DSC measurements were performed on a Microcal VP-DSC ultrasensitive differential scanning microcalorimeter (GE Healthcare Life Sciences, Piscataway, NJ, USA). Experiments were carried out at different concentrations of the protein from 1.25 to 10 µM, and at different scan rates from 0.5 to 1.5 K/min for each protein concentration. Before the measurements, the sample and reference solutions were properly degassed in an evacuated chamber attached to the calorimeter for 5 min at room temperature, and carefully loaded into the cells (each 0.5 ml) to avoid bubble formation. Exhaustive cleaning of the cells was undertaken before each experiment. An over-pressure of 1.8 kg/cm^2^ was always kept over the liquid in the cells throughout the scans to prevent any degassing during heating. The buffer scan (i.e., instrumental baseline) was determined before each sample scan, by filling both the sample and reference cells with the buffer used for the protein sample and using the same scanning parameters. For the experiments at different scan rates, 20 mM sodium phosphate buffer, which contained 50 mM NaCl and 50 mM imidazole at pH 7.4, was used. The reversibility of the thermal transition of MalZ was tested by checking the reproducibility of the calorimetric trace during the second heating of the sample after cooling it back to 10°C. Before the start of the first scan and between repeated scans, the solutions in the cells were allowed to equilibrate for 15 min at 10°C. The excess heat capacity curves were obtained from the calorimetric profiles by subtracting the buffer scan, normalizing the subtracted profiles by the protein concentration, and further correcting the profiles thus obtained with the suitable baseline positions, which were obtained by selecting appropriate pre- and post-transitional segments. The excess heat capacity profiles were smoothed and plotted using the Windows-based software package (Origin 7) provided with the instrument.

### CD measurements

CD measurements were performed on a Jasco-815 spectropolarimeter (Jasco International Co., Ltd., Tokyo, Japan), using a spectral band width of 2 nm and a cell path-length of 1.0 mm. The spectropolarimeter was equipped with a PTC-423S/15 thermocouple for temperature regulation. The peptide CD spectra were measured down to 225 nm, because the presence of 50 mM imidazole as a pH buffer produced strong light absorption and prohibited the measurements below 225 nm. All the CD spectra were background-corrected, smoothed, and converted to a mean residue ellipticity, [*θ*] = *M*_res_·*Θ*_obs_/*l*/*p* (deg·cm^2^/dmol), where *M*_res_ is the mean residue molecular weight, *Θ*_obs_ is the ellipticity measured in millidegree, *l* is the optical path-length in millimeter, and *p* is the protein concentration in mg/ml. For all the measurements, the buffer composition used was the same as in the DSC measurements. The thermal denaturation of MalZ was monitored by following the changes in ellipticity at 230 nm over a temperature range from 20 to 65°C. The experiments were carried out at different scan rates from 0.5 to 1.5 K/min at a protein concentration of 2.5 µM.

### UV-absorption (turbidity) measurements

UV-absorption measurements were performed in a Beckman-Coulter DU 800 UV-visible spectrophotometer with a spectral band width of 1.8 nm or less using matched 1.0-cm optical path-length quartz cuvettes. The spectrophotometer was equipped with a Peltier system, which provided a temperature controller for the multiple cell units, so that the absorption measurements could be performed directly as a function of temperature. The thermal denaturation of MalZ was monitored by recording the difference absorption at 340 nm over a temperature range from 20 to 65°C. Because the protein does not have any light absorption at this wavelength, the apparent absorption thus measured reflected solution turbidity. The experiments were carried out at different scan rates from 0.5 to 1.5 K/min at a protein concentration of 0.75 µM. We also carried out the experiments at different protein concentrations from 0.38 to 1.25 µM at a scan rate of 1.0 K/min. For all the measurements, the buffer composition used was the same as in the DSC measurements. The reversibility of the thermal transition was tested by cooling down the denatured protein at 65°C to 20°C, keeping the protein at 20°C for 10 min, and performing a rescan to check whether the same denaturation profile was regenerated.

### Data analysis

The DSC, CD and the difference absorption data were analyzed by the nonlinear least-squares method using the Origin 8 software package (OriginLab Corp.), by which we could perform global-fitting analysis. We coded the functions given by Eqs. (4) and (5) using the Origin C programming language, and used them for the data analysis.

## Results

### Irreversible thermal denaturation of MalZ

**Figure 1:**
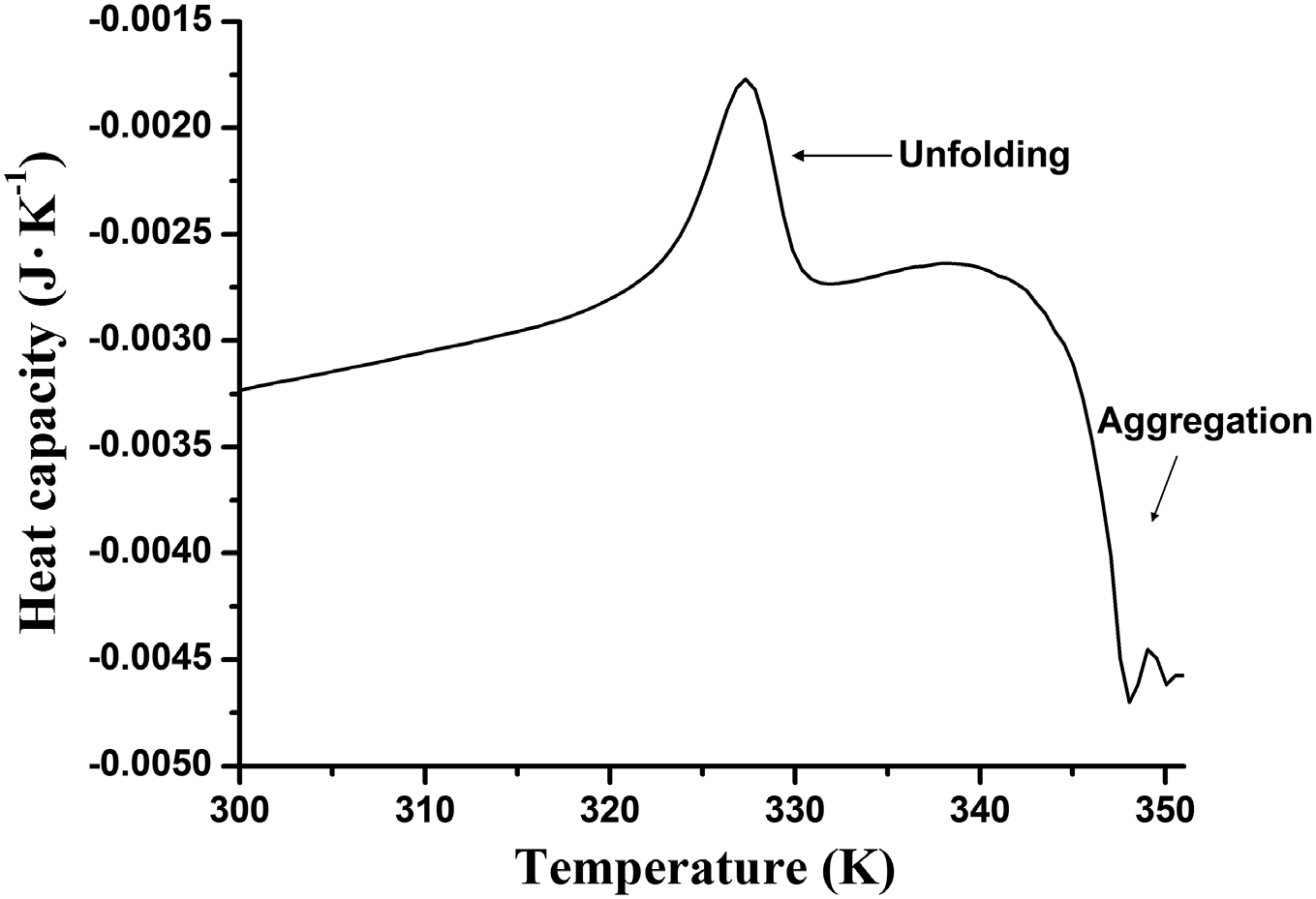
A raw DSC heating scan of MalZ in a 20 mM sodium phosphate buffer that contained 50 mM NaCl and 50 mM imidazole, at pH 7.4. The scan rate was 1.0 K/min, and the protein concentration was 10 µM. The profile shows an endothermic unfolding peak at 327 K, followed by a typical exothermal trace caused by protein aggregation above 337 K. The DSC scan shown is that before baseline subtraction.

Fig. 1 shows a raw DSC profile of MalZ at a scan rate of 1.0 K/min. The profile of the plot of excess heat capacity against temperature (*T*) displays an asymmetric, sharp endothermic peak with the temperature of the maximum heat capacity (*T*_max_) at 327.1 K (53.9°C) for this scan rate. When the protein sample was heated up to 353.3 K (80°C) and immediately cooled down to 10°C, the reheating scan demonstrated no thermal effect, indicating the irreversibility of the thermal transition. Furthermore, we observed an exothermal aggregation above 338 K (65°C); protein aggregation at a high temperature after completion of conformational unfolding often accompanies the exothermal effect [26]–[31]. To investigate whether the irreversibility was caused by the exothermal aggregation, we carried out a reheating scan, in which the protein was heated up to 331 K (58°C), where the unfolding of the protein was just completed but the exothermal aggregation had not yet taken place, and then cooled down to 10°C for reheating; the first heating and the reheating were carried out at the same scan rate (1.0 K/min). Again no thermal effect was observed. We thus concluded that the thermal denaturation of MalZ was calorimetrically irreversible.

**Figure 2:**
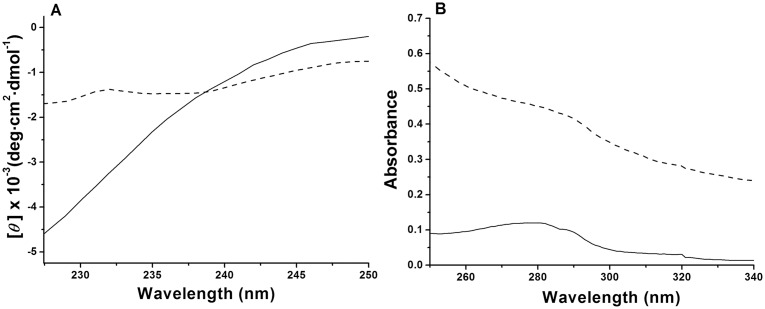
The CD (A) and UV-absorption (B) spectra of native (solid line) and thermally denatured (dashed line) MalZ at 20°C and 58°C, respectively. The solution condition was in 20 mM sodium phosphate buffer, containing 50 mM NaCl and 50 mM imidazole, at pH 7.4. The protein concentration used was 2.5 µM for CD measurement and 0.5 µM for UV-absorption measurements.

The thermal denaturation of MalZ was also studied by CD and UV-absorption spectroscopy. The CD was used to monitor changes in the secondary structure, and the UV absorption was used to measure solution turbidity (see below). Fig. 2(A) shows the far-UV CD spectra of MalZ in the native (N) (solid line) and the thermally denatured (F) (dashed line) states. The negative CD ellipticity observed in the N state markedly decreased below 238 nm in the F state, indicating the unfolding of the secondary structure by the thermal denaturation. However, we also observed a marked increase in the dynode voltage of the CD instrument when measuring the spectrum in the F state. The MalZ solution after the thermal denaturation became turbid, and hence the increase in the dynode voltage was caused by protein aggregation [19]. This aggregation was, however, different from the exothermal aggregation observed by DSC above 65°C, because the CD spectrum in the F state was measured at 58°C (Fig. 2(A)). Fig. 2(B) shows the UV-absorption spectra of MalZ in the N (solid line) and the F (dashed line) states. As expected, the apparent absorbance was markedly higher in the F state than in the N state. The increase in the absorbance was thus due to the turbidity production caused by aggregation.

### Effect of protein concentration

**Figure 3:**
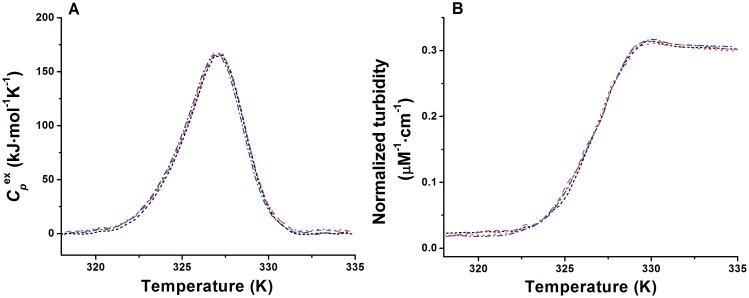
Temperature dependence of the molar excess heat capacity (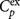) (A) and the normalized turbidity at 340 nm (B) at different concentrations of MalZ. (A) 1.25 µM (short dashed line (black)), 2.5 µM (dash dotted line (blue)), 5 µM (dotted line (red)) and 10 µM (dashed line (magenta)); and (B) 0.38 µM (short dashed line (black)), 0.75 µM (dash dotted line (blue)), 1 µM (dotted line (red)) and 1.25 µM (dashed line (magenta)). The scan rate was 1.0 K/min.

Because the occurrence of aggregation during the thermal denaturation of MalZ was evident, we investigated whether or not this aggregation significantly affect the DSC profiles of MalZ. We thus recorded the DSC thermograms given by temperature dependence of molar excess heat capacity (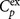) at various protein concentrations from 1.25 µM to 10 µM, and the results are shown in Fig. 3(A). Rather surprisingly, varying the protein concentration had no effects on the shape and height of the endothermic peak of thermal denaturation, and hence, there was no significant change in *T*_max_ with the protein concentration. Interestingly, the same results were also obtained in the UV-absorption-based experiments by taking the thermal scans from 20°C to 65°C at various protein concentrations. Fig. 3(B) illustrates the normalized turbidity, obtained by dividing the apparent absorption value at 340 nm by protein concentration in µM, plotted as a function of temperature. The experimental conditions used were the same as those in the DSC experiments, and the half-transition temperature (*T*_1/2_), where a half of the protein molecules were denatured, did not show any significant dependence on the protein concentration. It is thus concluded that the rate-limiting step of the thermal denaturation of MalZ is a mono-molecular process to which neither oligomerization nor aggregation contributes. The turbidity production caused by aggregation may take place after the rate-limiting step of the thermal denaturation.

### Scan-rate dependence

**Figure 4:**
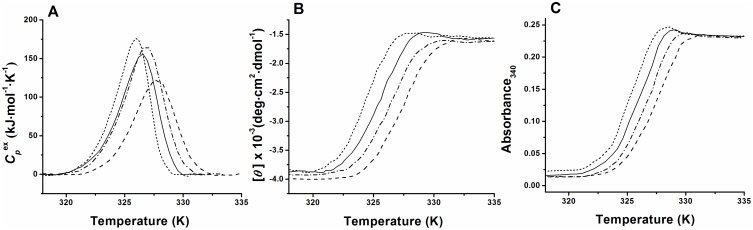
Temperature dependence of 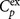 (A), the CD ellipticity at 230 nm (B) and the UV absorption at 340 nm (C) of MalZ at different scan rates. The scan rate were 0.5 K/min (short dashed line), 0.75 K/min (solid line), 1.0 K/min (dash dotted line) and 1.5 K/min (dashed line). The protein concentration was 1.25 µM for DSC measurements, 2.5 µM for CD measurement, and 0.75 µM for UV absorption measurements.

Because the thermal denaturation of MalZ is irreversible, it is important to investigate whether or not the denaturation is under kinetic control, and this can be tested by recording the DSC thermograms at different scan rates [5], [42]. We thus performed the DSC experiments at four different scan rates from 0.5 K/min to 1.5 K/min, and the results are shown in Fig. 4(A). As can be seen, the *T*_max_ and the shape of the thermogram changed with the scan rate, and as the scan rate increased, *T*_max_ shifted towards a higher temperature, indicating that the MalZ denaturation was kinetically controlled (Table 1). It has been shown that even a simple two-state reversible transition can behave irreversibly when an unfavorable combination of the scan rate, and the rate constant and the activation energy of folding-unfolding occurs [46], [47]. However, this possibility was excluded, because the MalZ thermal denaturation was an irreversible process as indicated by the absence of any thermal effect in reheating scan (see above).

**Table 1:** *T*_max_ and *T*_1/2_ values of the MalZ thermal denaturation at different scan rates.

	DSC	CD ellipticity	UV absorption
Scan rate (K/min)	*T*_max_ (K)	*T*_1/2_ (K)	*T*_1/2_ (K)
0.50	326.0	324.5	325.2
0.75	326.6	325.5	326.0
1.00	327.1	326.1	326.6
1.50	327.6	327.2	327.4

We also investigated the scan-rate dependence of the thermal denaturation monitored by CD ellipticity and UV absorption. The thermal transition curves were thus obtained by monitoring the ellipticity at 230 nm and the UV absorption at 340 nm as a function of temperature (Fig. 4(B) and (C)), at the same four different scan rates and under the same solution conditions as used in the above DSC experiments. As shown in Fig. 4(B) and (C), the CD-ellipticity-monitored and the UV-absorption-monitored thermal transitions were both changed with the scan rate in a very similar manner. The *T*_1/2_ increased with an increase in the scan rate, and the *T*_1/2_ values obtained by the CD and UV-absorption measurements were well coincident with each other (Table 1). Furthermore, the *T*_1/2_ values observed here were a little lower than but still nearly coincident with the *T*_max_ values observed in the DSC measurements (Table 1); because the DSC thermograms were asymmetric, *T*_1/2_ was a little lower than *T*_max_. These coincidences thus provide clear evidence that the endothermic thermograms of MalZ measured by DSC are associated with the protein unfolding transitions measured by the CD ellipticity and the solution turbidity transitions measured by the UV absorption (Fig. 4).

It should be noted that the turbidity transitions measured by the UV absorption were well coincident with the unfolding transitions measured by the CD ellipticity and that the apparent UV absorption (turbidity) in the post-transition region was weakly dependent on temperature and independent of the scan rate (Fig. 4(C)). This indicates that the turbidity production in the F state was not caused by nonspecific aggregation of denatured protein, but the protein aggregate in the F state had a definite size and a definite number of protein molecules, and hence the turbidity measured by UV absorption was proportional to the degree of denaturation. In fact, the protein solution after the thermal denaturation was opalescent but homogeneous, and no precipitates were observed under the conditions of Fig. 4.

### Analysis of the thermal denaturation curves

The irreversible thermal denaturation of proteins is usually discussed in terms of the Lumry-Eyring model [41], [42]:
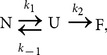
(2)where U is the unfolded state, reversibly produced from N but irreversibly altered into F, *k*_1_ and *k*_−1_ are the forward and backward microscopic rate constants between N and U, and *k*_2_ is the rate constant for the irreversible alteration. Therefore, this model includes two steps: (i) the reversible conformational unfolding between N and U, and (ii) the irreversible alteration from U to F.

However, in many cases, the irreversible thermal denaturation of proteins as monitored by DSC or other techniques can be phenomenologically described by a simple one-step irreversible model (Eq. (1)), and the temperature dependence of the first-order rate constant *k* in Eq. (1) is represented by the Arrhenius equation [5]–[11]:
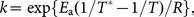
(3)where *E*_a_ is the activation energy, *R* is the gas constant, and T* is the temperature at which *k* is equal to 1 min^−1^. Theoretically, this one-step irreversible model is regarded as a limiting case of the Lumry-Eyring model (Eq. (2)) [5]–[7] (see DISCUSSION). Therefore, it does not necessarily mean that N directly converted to F. We used the one-step irreversible model for analysis of the present DSC data (Fig. 4(A)) and the data monitored by the CD ellipticity and the UV absorption (Fig. 4(B) and (C)).

#### DSC data

**Figure 5:**
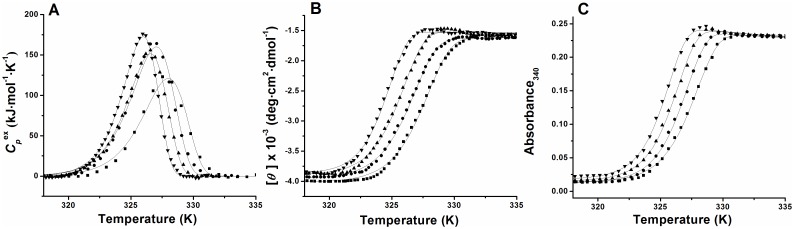
Theoretical fitting of the DSC profiles (A), the thermal unfolding profiles obtained by CD ellipticity at 230 nm (B), and the turbidity transition profiles obtained by UV absorption at 340 nm (C) of MalZ at different scan rates. The scan rates were 0.5 K/min (▾), 0.75 K/min (▴), 1.0 K/min (•) and 1.5 K/min (▪). The continuous lines indicate the theoretical fitting curves based on Eq. (4) (A) and Eq. (5) (B) and (C). The experimental data were the same as those in Fig. 4, and the representative data points are shown by the symbols.

For the one-step irreversible denaturation of a protein (Eq. (1)), the excess heat capacity 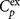 recorded in a DSC scan is given by the following equation [48]:
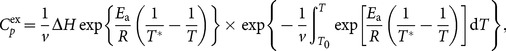
(4)where *ν* is the scan rate given by *v* = d*T*/d*t* (K/min), Δ*H* is the enthalpy difference between the F and the N states, and *T*_0_, which was set at 315 K, is the initial temperature for integration; *T*_0_ should be set at a temperature where the protein is fully in the N state. Therefore, we carried out non-linear least-square analyses on the DSC data at four different scan rates by fitting the data to this equation either individually or globally. In the global analysis, Δ*H*, *E*_a_ and T* were global parameters common among the data at different scan rates, and *ν* was used as an additional variable. The best-fit parameter values for Δ*H*, *E*_a_ and T*are summarized in Table 2, and the solid lines through the data points in Fig. 5(A) were theoretical curves obtained by using the parameter values from the individual fits in Table 2. It should also be noted that in the analysis of irreversible thermal transition by DSC, we focused on the kinetics represented by the Arrhenius equation (Eq. (3)), and hence assumed that the Δ*H* was independent of *T* in a narrow temperature range of the thermal transition [48].

**Table 2:** Arrhenius-equation parameters obtained by the non-linear least-squares fitting of the DSC data (Fig. 4(A)) to Eq. (4) *.

	Temperature scan rate (K/min)				
	0.50	0.75	1.00	1.50	Global fit
*E*_a_ (kJ/mol)	635.5±2.5	571.5±3.3	528.0±6.7	518.4±11.7	571.1±5.0
*T* * (K)	327.4±0.01	327.7±0.01	327.9±0.04	328.2±0.06	327.7±0.03
Δ*H* (kJ/mol)	646.4±4.1	633.5±4.6	725.5±11.7	561.5±13.8	650.6±7.9

*Error values shown are fitting error estimates in the least-squares analysis.

As seen from Fig. 5(A), the theoretical curves are in excellent agreement with the experimental data, and from Table 2, the theoretical estimates at different scan rates are in reasonable agreement with each other and also with those obtained by the global fit. Therefore, as a first-order approximation, the simple one-step irreversible model (Eq. (1)) well represents the thermal denaturation of MalZ. An attempt to fit the present data to a more complicated two-step model (Eq. (2)), in which the rapid pre-equilibrium between N and U was assumed to take place before the second irreversible step from U to F, by using an equation formulated by Sanchez-Ruiz [42] (Eq. (13) in ref. [42]) did not improve the fitting results, indicating that the simplest one-step irreversible model (Eq. (1)) was sufficient to describe the MalZ denaturation quantitatively.

##### CD and UV-absorption data

For the one-step irreversible denaturation (Eq. (1)), a physical quantity used to follow the denaturation, *y*, which is linearly related to the extent of denaturation, is given by the following equation [48]–[50]:
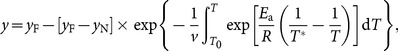
(5)where *y*_N_ and *y*_F_ are the *y* values in the pure N state and the irreversibly denatured F state, respectively, and we assume that these values linearly depend on temperature as *y*_N_ = *a*_1_+ *a*_2_ *T* and *y*_F_ = *b*_1_+ *b*_2_ *T*, where *a*_1_, *a*_2_, *b*_1_ and *b*_2_ are constants. We carried out non-linear least-square analyses on the denaturation curves monitored by the CD ellipticity and the UV absorption at four different scan rates by fitting the denaturation curves to Eq. (5) either individually or globally. In the global analysis, *E*_a_ and T* were global parameters common among the different denaturation curves, while *a*_1_, *a*_2_, *b*_1_ and *b*_2_ were local parameters for each denaturation curve, and *ν* was used as an additional variable. The best-fit parameter values for *E*_a_ and T*are summarized in Table 3, and the solid lines through the data points in Fig. 5(B) and (C) were theoretical curves obtained by using the parameter values from the individual fits in Table 3. All the theoretical curves are again in excellent agreement with the experimental data. The parameter values for the CD ellipticity and the UV absorption listed in Table 3 are in excellent agreement with each other, and they are also in excellent agreement with those in Table 2 obtained from the DSC data. The results thus strongly demonstrate that the irreversible denaturation observed by the DSC experiments was coincident with the irreversible conformational unfolding of MalZ monitored by the CD ellipticity and also with the irreversible turbidity production monitored by the UV absorption.

**Table 3:** Arrhenius-equation parameters obtained by the non-linear least-squares fitting of the far-UV CD data (Fig. 4(B) and the UV-absorption data (Fig. 4(C)) to Eq. (5) *.

	Temperature scan rate (K/min)				
	0.50	0.75	1.00	1.50	Global fit
Far-UV CD at 230 nm					
*E*_a_ (kJ/mol)	617.2±6.9	557.8±6.1	514.1±4.9	508.0±5.8	554.2±4.9
*T* * (K)	326.5±0.05	327.2±0.05	327.6±0.04	328.1±0.04	327.3±0.04
UV absorption (turbidity) at 340 nm					
*E*_a_ (kJ/mol)	628.4±5.4	599.6±4.3	579.5±5.7	540.3±5.9	578.3±3.5
*T* * (K)	327.2±0.04	327.5±0.04	327.8±0.04	328.1±0.05	327.6±0.03

*Error values shown are fitting error estimates in the least-squares analysis.

### Validity of the one-step irreversible model

**Figure 6:**
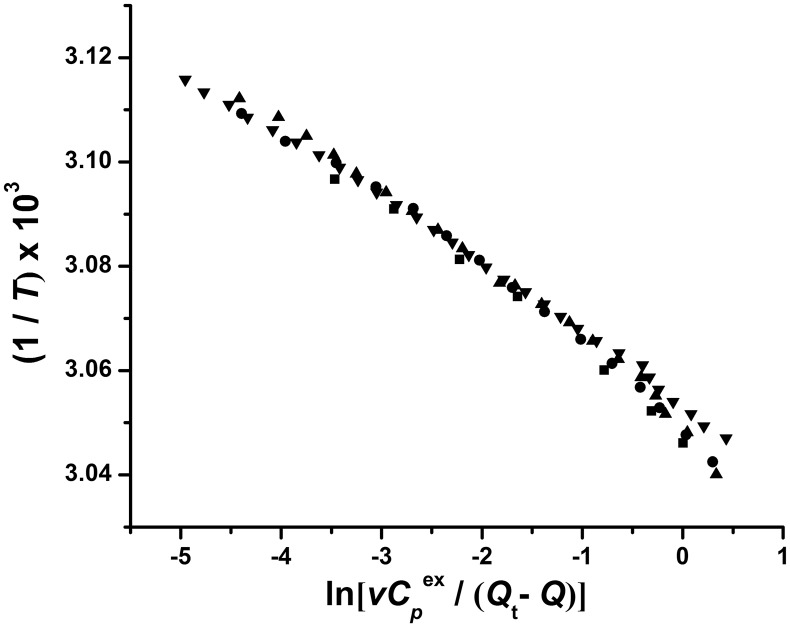
Dependence of 1/*T* on 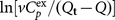 for MalZ (Eq. (6)). The symbols are the same as in Fig. 5 and indicate different scan rates.

Kurganov *et al*. [48] have proposed a very useful criterion for the validity of the one-step irreversible model of the thermal denaturation of proteins, and this is based on the construction of the linear anamorphosis of the DSC curves at different scan rates in the coordinates {1/*T*; 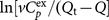} according to the equation:
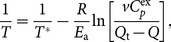
(6)where *Q* is the heat absorbed during heating of the protein to the temperature *T*, and *Q*_t_ is the total heat absorbed through the denaturation process, and hence equivalent to the calorimetric enthalpy change Δ*H* in the one-step irreversible model. If the model is valid, the points corresponding to all the scan rates should lie on a common straight line. Fig. 6 shows the 1/*T* vs. 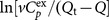 plots thus obtained by using the *v* and Δ*H* ( = *Q*_t_) values of the individual fits shown in Table 2. As seen from Fig. 6, the above criterion is satisfactorily fulfilled for MalZ, further demonstrating the validity of the one-step irreversible model for the thermal denaturation. From the slope and the intercept of the plots in Fig. 6, we obtained the *E*_a_ and T* values by linear regression analysis of all the data together, and the values thus obtained were *E*_a_ = 579.6±6.8 kJ/mol and T* = 327.1±0.04 K. These values were in good agreement with the corresponding values in Tables 2 and 3.

## Discussion

We studied the thermal denaturation of *E. coli* MalZ by DSC, CD-ellipticity and UV-absorption measurements; DSC was used to monitor the heat absorption, CD to monitor the conformational unfolding, and UV absorption to monitor the turbidity production. The thermal denaturation was irreversible, because there were no thermal effects in reheating scans after the thermal transition in any of the three measurements, although the physical parameters monitored by these measurements were different. The lack of a protein concentration effect on *T*_max_ and *T*_1/2_ as well as on the shape of the DSC thermograms and the UV-absorption-based transition curves indicated that the denaturation was rate-limited by a mono-molecular process (Fig. 3). However, the thermal denaturation was coupled with protein aggregation that produced the turbidity as detected by the UV absorption (Fig. 4(C)), and this aggregation was different from the exothermal aggregation observed by DSC at a higher temperature above 65°C (Fig. 1). From the DSC thermograms and the transition curves measured by the CD ellipticity and the UV absorption, which were recorded at four different scan rates from 0.5 K/min to 1.5 K/min (Fig. 4), the *T*_max_ and *T*_1/2_ depended significantly on the scan rate and shifted to a higher temperature with an increase in the scan rate, indicating that the thermal denaturation of MalZ was kinetically controlled [5], [42]. In the following, we further discuss the thermal denaturation of MalZ in terms of the one-step irreversible model, kinetic stability and the Arrhenius-equation parameters, and finally the molecular mechanisms of the irreversible thermal denaturation.

### The one-step irreversible model

The thermal denaturation of MalZ was well represented by the one-step irreversible model (Eq. (1)). We analyzed the DSC and the spectroscopic data (CD ellipticity and UV absorption (turbidity)) on the basis of this model, with the use of Eqs. (4) and (5), respectively, by the non-linear least-squares method, and the theoretical curves thus obtained exhibited excellent agreement with the experimental data (Fig. 5), indicating the validity of the one-step model. The values of parameters obtained by the global fitting of the data at different scan rates were in agreement with those obtained by the individual fittings (Tables 2 and 3). The Arrhenius-equation parameters (*E*_a_ and T*) obtained from the DSC, CD-ellipticity and UV-absorption data were all in excellent agreement with each other (Tables 2 and 3), clearly indicating that the thermal denaturation was closely coupled with the conformational unfolding monitored by the CD ellipticity, as well as the turbidity production monitored by the UV absorption. The plots of 1/*T* vs. 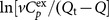 at different scan rates gave a common straight line, satisfying a criterion for the one-step irreversible model and further strengthening the validity of the model [6], [48], [49], [51]–[53] (Fig. 6). Nevertheless, the one-step irreversible model predicts that, while the *T*_max_ and *T*_1/2_ should be strongly scan-rate dependent, the shape of the DSC transitions should change comparatively little with scan rate [5], [8], [42]. The data in Fig. 4(A) and (B), however, indicate some significant change in shape, and this is reflected in a clear trend in the *E*_a_ values in Tables 2 and 3. Therefore, it should be noted that the one-step irreversible model may only provide an approximate, first-order description of the thermal denaturation kinetics of MalZ.

The one-step model of the irreversible thermal denaturation of proteins is a phenomenological one, and it is regarded as a limiting case of the more general Lumry-Eyring model (Eq. (2)) [41]. There are two limiting cases, in which the Lumry-Eyring model is reduced to the one-step model, depending on whether the U state in Eq. (2) prefers to refold to N or to undergo the irreversible alteration to F [7], [42]. Thus, when *k*_−1_>>*k*_2_ and the equilibrium constant between N and U, *K* ( = *k*_1_/*k*_−1_), is much less than unity in Eq. (2), the apparent rate constant *k* in Eq. (1) is given by *k* ≈*Kk*_2_, and hence the activation energy *E*_a_ corresponds to the sum of the enthalpy change for conformational unfolding (N↔U) and the activation energy of the irreversible alteration (U→F). On the other hand, when *k*_2_>>*k*_−1_, the conformational unfolding is rate-limiting (*k*≈*k*_1_), and hence the values of *E*_a_ correspond to the activation energy of unfolding. As will be shown later, a consideration of the molecular basis of the irreversibility demonstrates that the latter is the mechanism of the MalZ thermal denaturation (see below).

### Kinetic stability and the Arrhenius-equation parameters

Over the last 25 years, a large number of *in vitro* and irreversible, protein thermal denaturation processes have been reported, and most of the denaturation processes so far reported have been well represented by the one-step model or closely related kinetic models [5]–[11]. Therefore, the one-step model, though phenomenological and a limiting case of the Lumry-Eyring model, is rather common for the irreversible thermal denaturation of proteins, and the half-life *τ*_1/2_, given by *τ*_1/2_ = (ln 2)/*k* at a physiological temperature (37°C), provides a measure of the kinetic stability of proteins. The kinetic stability of proteins is important for understanding protein stability *in vivo* [7], [8], [12], the molecular mechanisms of protein misfolding diseases [9], [11], [13], and natural selection for stability during protein evolution [14]–[16].

Table 4 summarizes the Arrhenius-equation parameters, including the half-life *τ*_1/2_, of the one-step irreversible thermal denaturation of different proteins, and the parameter values are compared with those for MalZ obtained in the present study. For all the proteins listed in Table 4, the DSC data at different scan rates were analyzed in a quantitative manner by Eq. (4) or an equivalent equation. A more comprehensive and earlier list of the parameters of the one-step irreversible denaturation of proteins up to the year of 1999 was reported by Lyubarev and Kurganov [6]. From Table 4, the activation energy *E*_a_ of MalZ is the second highest among the proteins. All the proteins except for glucose oxidase, lipase B, peroxidase isoenzyme *c* and rhodopsin have expected *τ*_1/2_ values larger than 10 days at 37°C, and the *τ*_1/2_ of MalZ is 68 days. Considering the doubling time (∼30 min) of *E. coli*, it is thus concluded that MalZ has sufficiently high kinetic stability against denaturation at a physiological temperature (37°C).

**Table 4:** Comparison of parameters of the one-step irreversible thermal denaturation of different proteins.

Protein	Source	*M*_w_(kDa)	Num. ofSubunits	pH	Δ*H* (kJ/mol)	*T* * (K)	*E*_a_ (kJ/mol)	*τ*_1/2_ (days)(at 37°C)	Ref.
Alanine:glyoxylateaminotransferase*	human	87.6	2	7.4	1112±46	350.8	443±13	2.1×10^5^	Pey *et al*. (2011) [9]
α-Amylase*	*Bacillus* *halmapalus*	56	1	8.0	2443±22	374	172±3	42	Nielsen *et al*. (2003) [54]
Creatinkinase	rabbitmuscle	84	2	8.0	1079±1	329.01±0.01	461.0±0.7	14	Lyubarev *et al*. (1999) [51]
Cry3A δ-endotoxin*	*Bacillus* *Thuringiensis*var. *tenebrionis*	67	1	3.5	1330	345.2	464	4.1×10^4^	Potekhin *et al*. (1999) [55]
Fimbrial DraEsubunit	uropathogenic*E*. *coli*	16	1	7.5	712.5±45.9	362.4±0.3	463.5±20.8	8.7×10^7^	Pia˛tek *et al*. (2009) [53]
GlutathioneS-transferase	*Schistosoma* *japonicum*	52	2	7.5	712.3±15.0	333.5±0.5	392.9±37.6	21	Quesada-Soriano *et al*. (2006) [30]
Glucoseoxidase*	*Aspergillus* *niger*	160	2	7.2	n.a.	332.7	280	0.8	Zoldák *et al*. (2004) [56]
Latex amine oxidase	*Euphorbia* *characias*	148	2	7.0	1981–2318	342.0–342.5	403–411	1.3×10^3^	Amani *et al*. (2007) [52]
Lectin	lentil	52	4	7.4	812–1056	353.6±0.09	357.8±1.3	1.2×10^4^	Marcos *et al*. (1999) [49]
Lipase B	*Candida rugosa*	60	1	7.2	996–1248	335.3–336.3	241.4–275.3	0.6–1.6	Shnyrov *et al*. (1999) [57]
Methionineaminopeptidase*	*Pyrococcus furiosus*	33	1	3.46	∼1400	371	325	4.2×10^5^	Potekhin *et al*. (2000) [58]
Nitritereductase	*Alcaligenes* *faecalis*	111	3	7	1627	374	518	3.8×10^11^	Stripe *et al*. (2005) [59]
Ovalbumin*	chicken	43	1	7.0	800	350.8	430	1.2×10^5^	Weijers *et al*. (2003) [60]
Peroxidase	african oil palmtree *Elaeis guineersis*	57†	n.a.	3.0	251–257	347.5±0.3	426.8±5.9	(3.1–8.3)×10^4^	Rodríguez *et al*. (2002) [61]
Peroxidase	royal palm tree	90	2	3.0	509–603	342.0±0.2	542.6±3.3	1.6×10^5^	Zomorano *et al*. (2008) [62]
Peroxidaseisoenzyme *c*	horseradish	44	1	3.0	n.a.	344.4±0.5	155.9±2.5	0.2	Pina *et al*. (2001) [63]
Phenylalaninehydroxylase*	*Chloroflexus* *aurantiacus*	32–34	1	7.0	300±38	352	235±23	19	Pey & Martinez (2009) [64]
Phosphoglyceratekinase 1*	human	45	1	7.4	656±75	325.6	798±79	1.2×10^3^	Pey *et al*. (2013) [11]
Rhodopsin* ‡	bovine	39	1	7.0	n.a.	326	386	1.6	Corley *et al*. (2011) [10]
Tetracyclinerepressor	*E. coli*	47	2	8.0	525.3±87.3	334.7±0.9	409.2±30.5	55	Kędracka *et al*. (2003) [65]
Xylanase	*Trichoderma reesei*	21	1	5.0	n.a.	335.2±0.2	455.2±5.0	258	Jänis *et al*. (2008) [31]
MalZ	*E. coli*	69	1	7.4	650.6±7.9	327.7±0.03	571.1±5.0	68	this study

*For these proteins, T* values were not reported, but instead, the *T*_max_, *E*_a_ and *v* values were given. The T* values were thus calculated by the relation: T* = (*RT*_max_^2^/*E*_a_)×ln (*RT*_max_^2^/*E*_a_ *v*) + *T*_max_. [5], [66].

† Determined by SDS polyacrylamide gel electrophoresis.

‡ Solubilized in 100 mM octy-β-D-glucopyranosid.

### Molecular mechanisms of the MalZ thermal denaturation

The most important feature of the MalZ thermal denaturation observed in the present study is the remarkable coincidence of the turbidity production, measured by UV absorption, with the thermal transitions measured by DSC and CD ellipticity. The turbidity production, though caused by protein aggregation, was different from the exothermal aggregation observed at a higher temperature above 65°C (Fig. 1). The exothermal aggregation, often observed in the irreversible thermal denaturation of proteins [26]–[31], occurs after the endothermic thermal transition and sometimes hampers quantitative analysis of the thermal transition [27], [30]. The turbidity production of MalZ, however, occurred concurrently with the endothermic thermal transition, and the Arrhenius-equation parameters (*E*_a_ and T*) obtained from the turbidity transition curves were in excellent agreement with those obtained from the DSC and CD measurements (Tables 2 and 3). In the post-transition region, the turbidity transition curves at different scan rates were superimposable and weakly dependent on temperature, as observed in the CD-measured transition curves (Figs. 4 and 5). It is thus strongly suggested that the protein aggregates, which produce the turbidity in the F state, may have a definite size and a definite number of protein molecules, and hence the turbidity measured by UV absorption is proportional to the degree of thermal denaturation. Furthermore, the thermal transitions measured by DSC and UV absorption were independent of protein concentration (Fig. 3), indicating that the denaturation was rate-limited by a mono-molecular process.

From the above results, we propose the following model, in which the irreversible thermal denaturation of MalZ is rate-limited by the conformational unfolding from N to U, and the irreversible step, producing the aggregates, is much faster than the transition between N and U, as


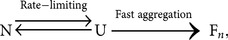
(7)

 (7)where the subscript *n* of F*_n_* indicates the aggregation number of MalZ in the F state. The enthalpy change Δ*H* of the whole reaction, as measured by DSC (Table 2), is thus given by the sum of the enthalpy change of the unfolding step (N→U) and the enthalpy change of the irreversible aggregation step (U→F*_n_*), but often the latter contribution is assumed to be negligibly small [7], [42].

It has been reported here that the irreversibly formed aggregates F*_n_* (Eq. (7)) remain preserved during thermal denaturation of MalZ. This phenomenon has also been observed in irreversible thermal denaturation of several proteins, including five homologous α-amylases [18], *E*. *coli* asparaginase-2 [19], human apolipoprotein C-1-dimyriostoil phosphatidylcholine complex [19] and amyloidogenic immunoglobulin light chain [20], where the irreversible thermal denaturation was studied by CD spectroscopy and turbidity (or light scattering). The unfolding and aggregation transition curves thus obtained showed excellent coincidence with each other for these proteins [18]–[20]. For α-amylases and amyloidogenic immunoglobulin light chain, the denaturation kinetics were studied at different protein concentrations, and the denaturation processes were independent of protein concentration [18], [20], the behavior identical to that observed here in the MalZ thermal denaturation. These results thus strongly suggest that the model of the irreversible thermal denaturation, rate-limited by conformational unfolding followed by rapid aggregation, of MalZ (Eq. (7)) is not exceptional, but may also be applied to the proteins mentioned above.

For many globular proteins, the irreversible thermal denaturation exhibits a typical asymmetric endothermic thermogram of DSC (see Figs. 3 and 4). Formation of aggregates has been considered a major cause of the irreversibility, but the detailed molecular mechanisms of aggregate production responsible for the endothermic thermal denaturation have not been well understood. The absence of the protein-concentration dependence of DSC thermograms has occasionally been taken as evidence for the absence of aggregation or the absence of change in the oligomerization state in oligomeric proteins [30], [59], [67]. The absence of the protein-concentration dependence in the irreversible thermal denaturation, however, does not necessarily mean the absence of aggregation itself; rather, it just means that the rate-limiting step of the denaturation is a mono-molecular process, and hence not influenced by aggregation. In this regard, it should be noted that the irreversible DSC thermogram (Eq. (4)) and the irreversible thermal transition curve (Eq. (5)) are both given as a function of the rate constant *k* (Eq. (3)). The aggregation may take place after the rate-limiting step as observed here in MalZ (Eq. (7)), and such aggregation can be monitored by the turbidity using UV absorption. The present study thus demonstrates that the turbidity measurements together with DSC and CD measurements in the irreversible thermal denaturation of proteins will provide a very effective approach for understanding the mechanisms of the irreversible denaturation of proteins, and further studies in this direction may be required to fully elucidate the irreversible thermal denaturation of proteins.

Although the *in vitro* thermal denaturation of MalZ is irreversible, it can refold into the N state *in vivo* and *in vitro* in the presence of the chaperonin GroEL/GroES complex, and the chaperonin-assisted folding of MalZ takes place in a *trans* mechanism [43]. Paul *et al.* [68] have also shown that not only the chaperonin complex but also chemical chaperones such as glycerol, dimethylsulfoxide and trimethylamine-*N*-oxide can also promote to a certain extent the spontaneous folding of MalZ. These chemical chaperones effectively inhibited protein aggregation, and hence enhanced the *in vitro* refolding of MalZ. The results are thus fully consistent with the present model (Eq. (7)), in which the conformational unfolding between N and U is reversible, and this is followed by the fast irreversible aggregation.

## Conclusions

The thermal denaturation of *E. coli* MalZ, studied by DSC, CD and UV-absorption (turbidity) measurements, was irreversible, and well represented by the one-step irreversible model. From comparison of the Arrhenius-equation parameters of MalZ with those of other proteins, for which the irreversible thermal denaturation was well characterized, MalZ was found to have sufficiently high kinetic stability with a half-life of 68 days at a physiological temperature. Although the MalZ denaturation was phenomenologically represented by the one-step model, the molecular mechanisms involved two steps: (1) the reversible conformational unfolding, which was rate-limiting for the whole process, followed by (2) the fast irreversible aggregation, which produced the solution turbidity.
